# Supplementary material for: Optimisation of Sample Preparation from Primary Mouse Tissue to Maintain RNA Integrity for Methods Examining Translational Control
Source: Cancers (Basel). 2023 Aug 5;15(15):3985. doi: 10.3390/cancers15153985 (PMC10417042; doi:10.3390/cancers15153985)

Main Fig. 2A

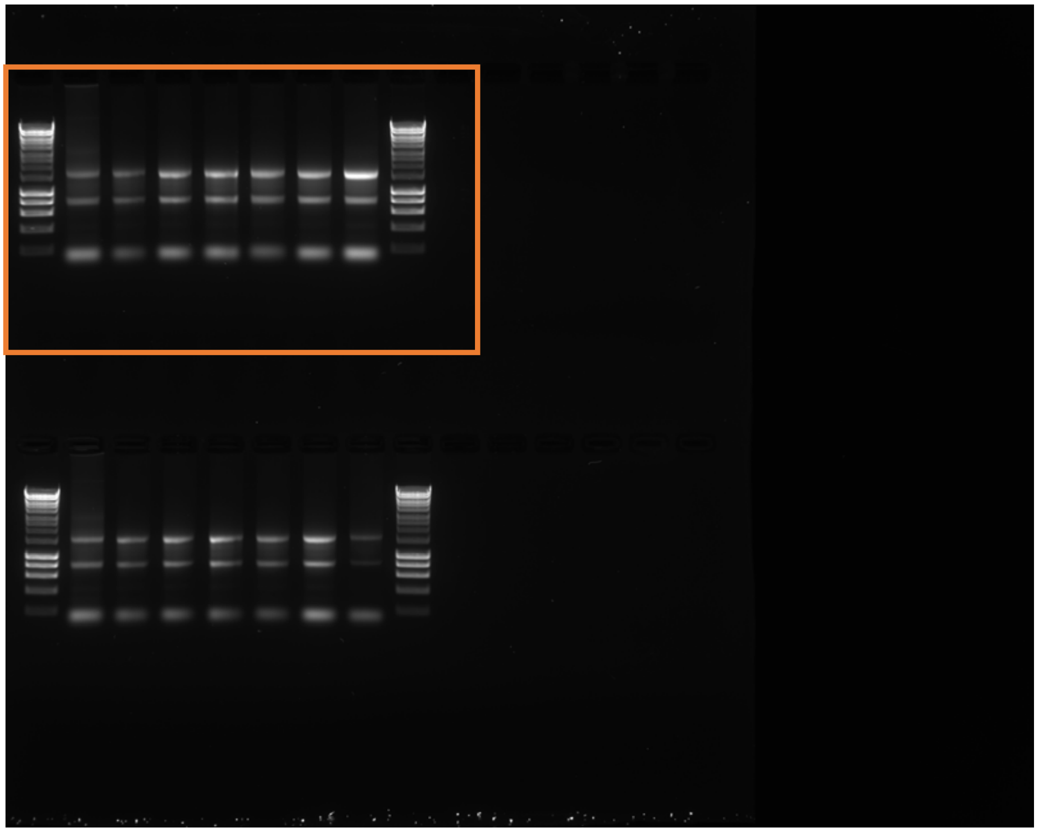

Main Fig. 2B

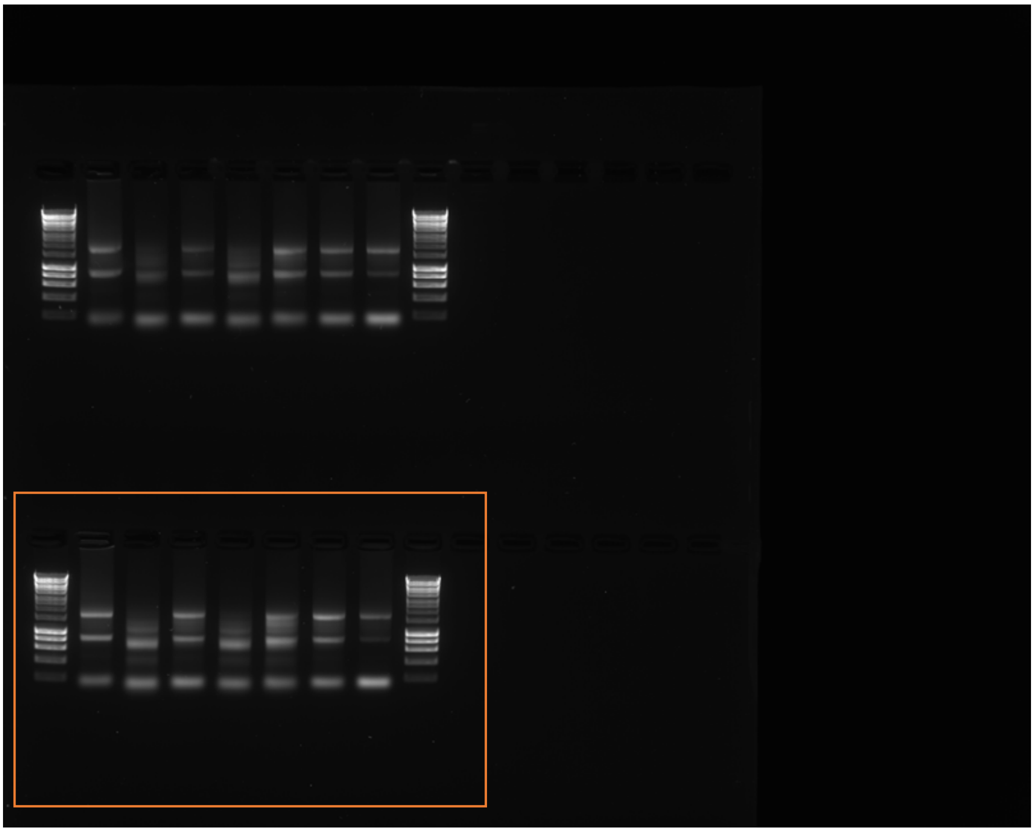

Main Fig. 2C

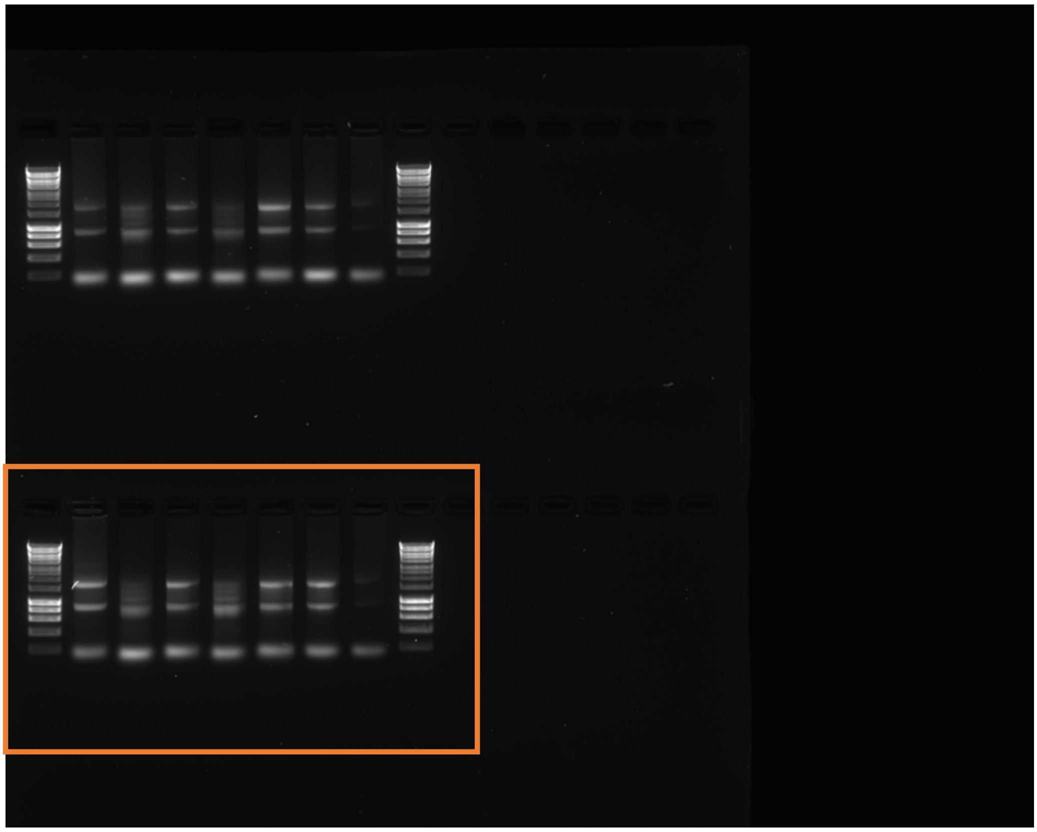

Main Fig. 2D

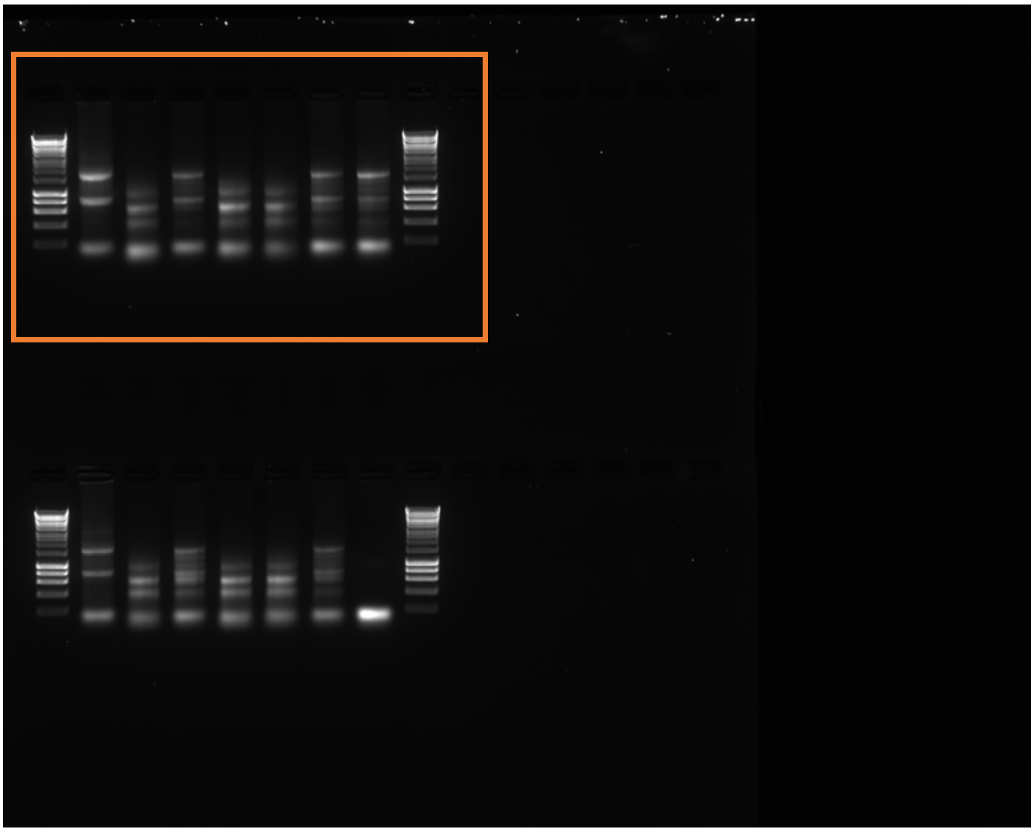

Main Fig. 2E

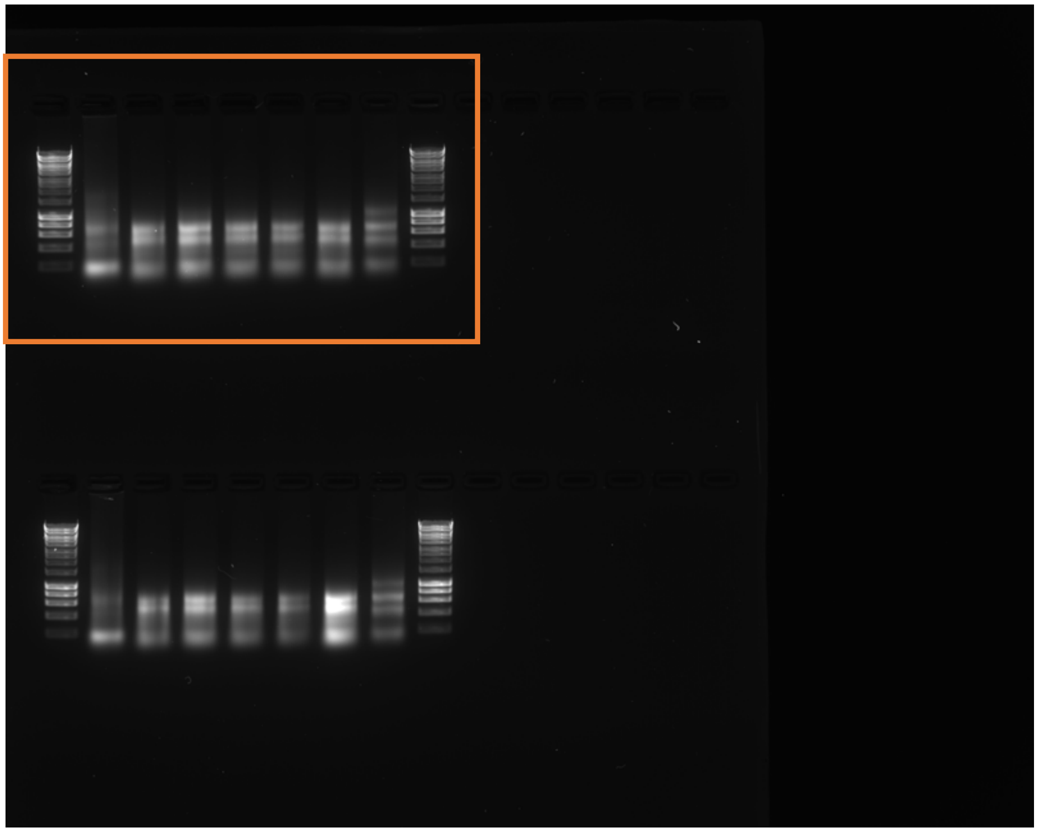

Main Fig. 2F

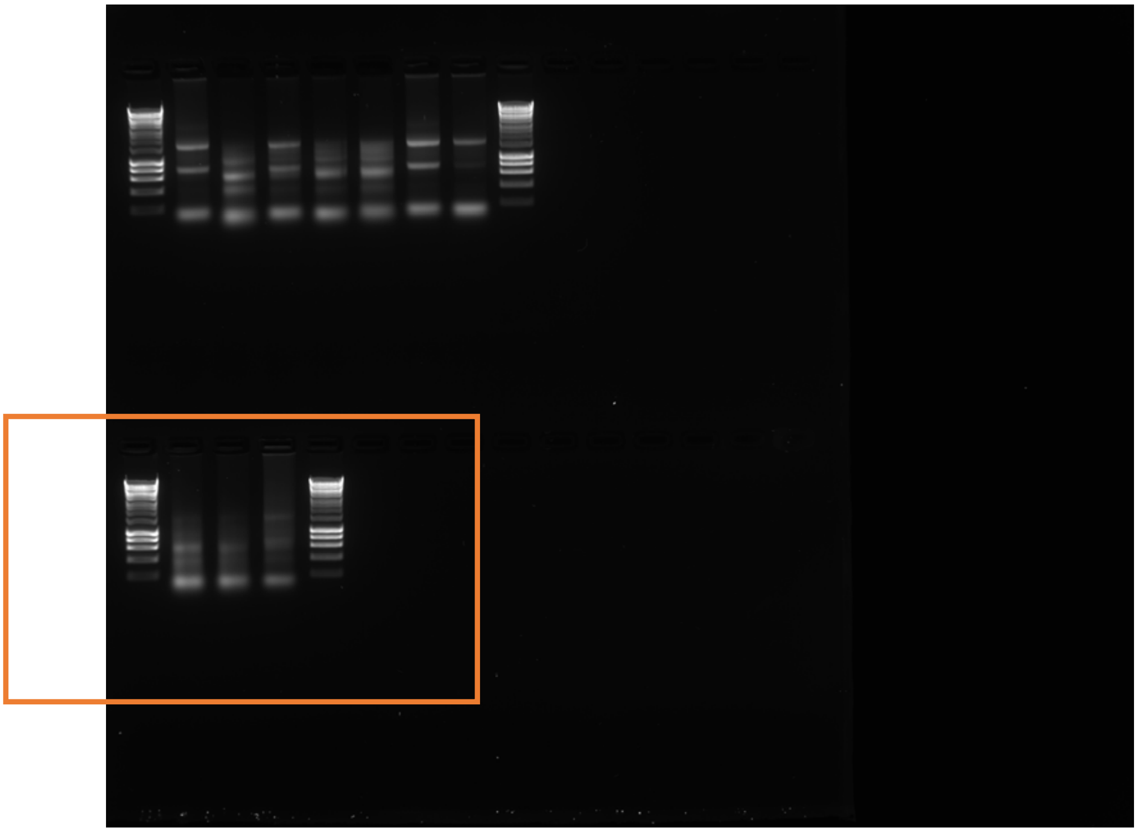

Main Fig. 2G

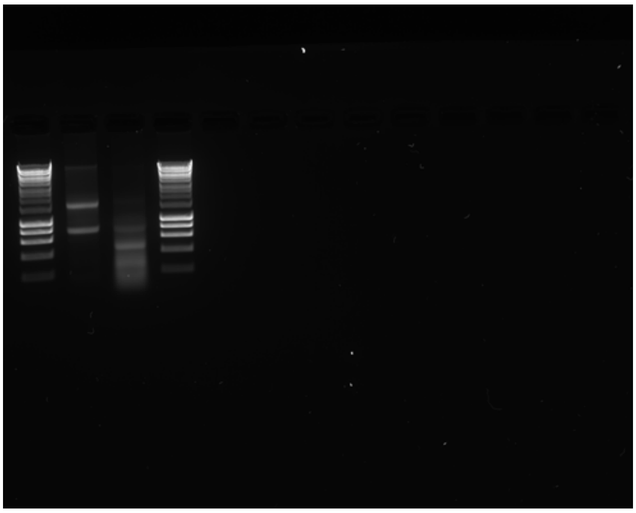

Main Fig. 2H

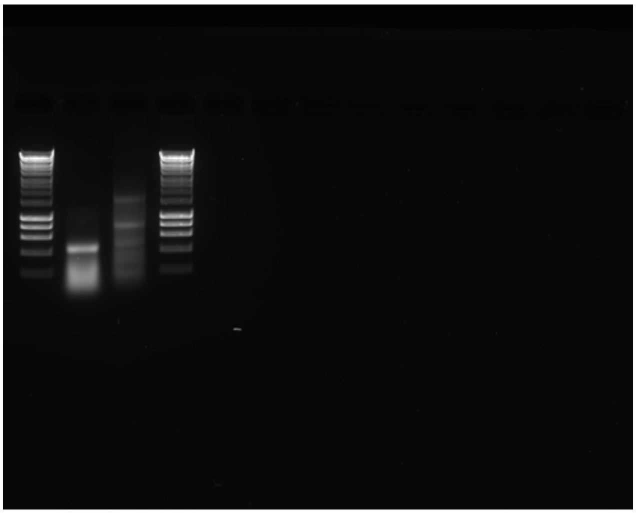

Main Fig. 3B

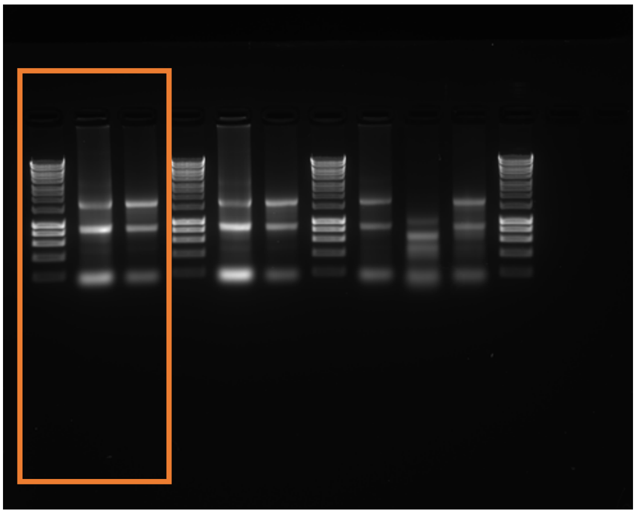

Main Fig. 3D

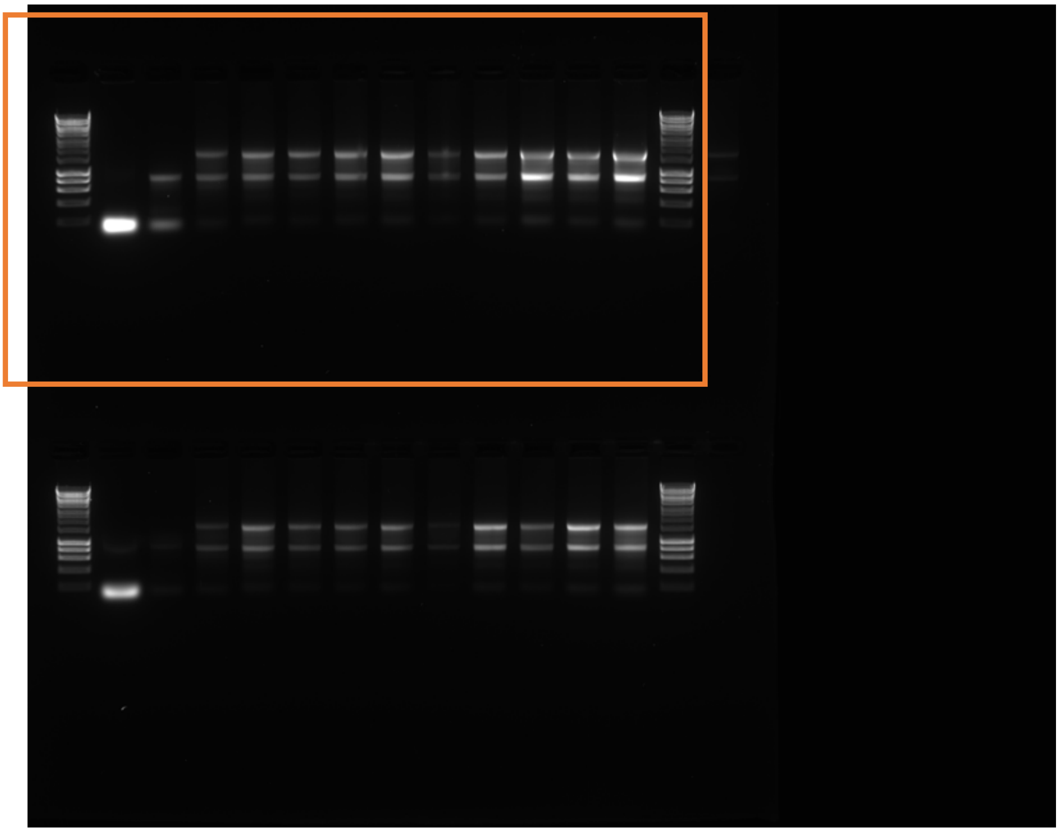

Main Fig. 4A

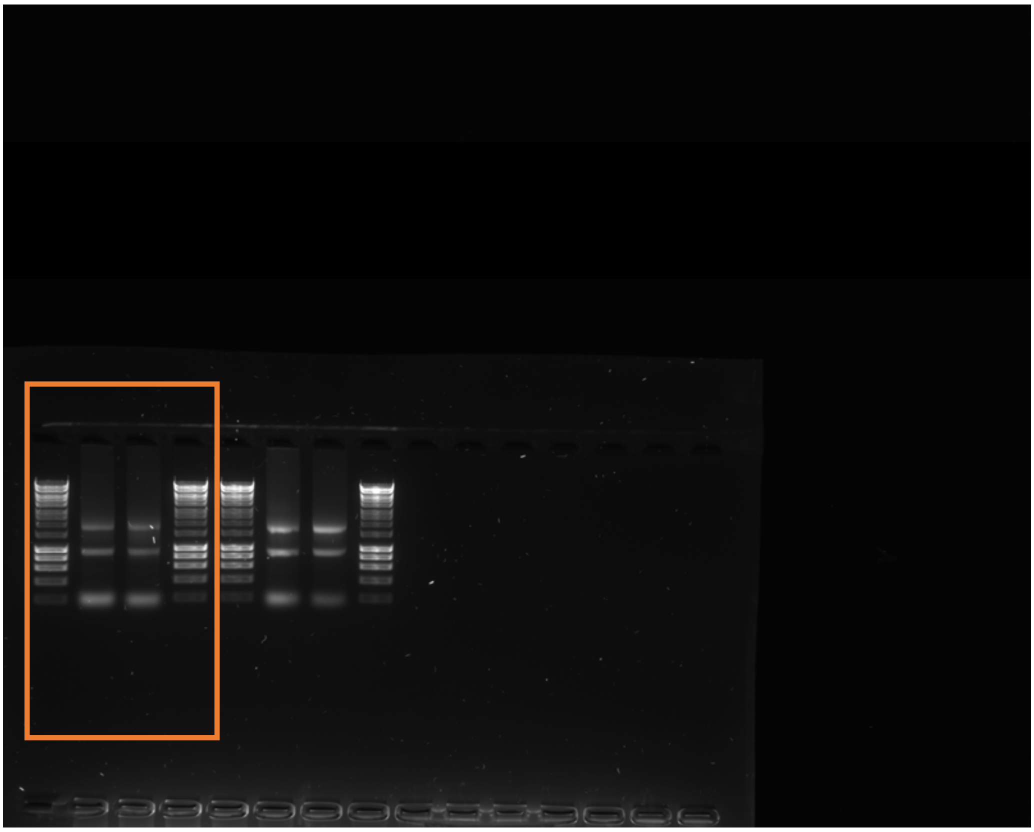

Main Fig. 4C

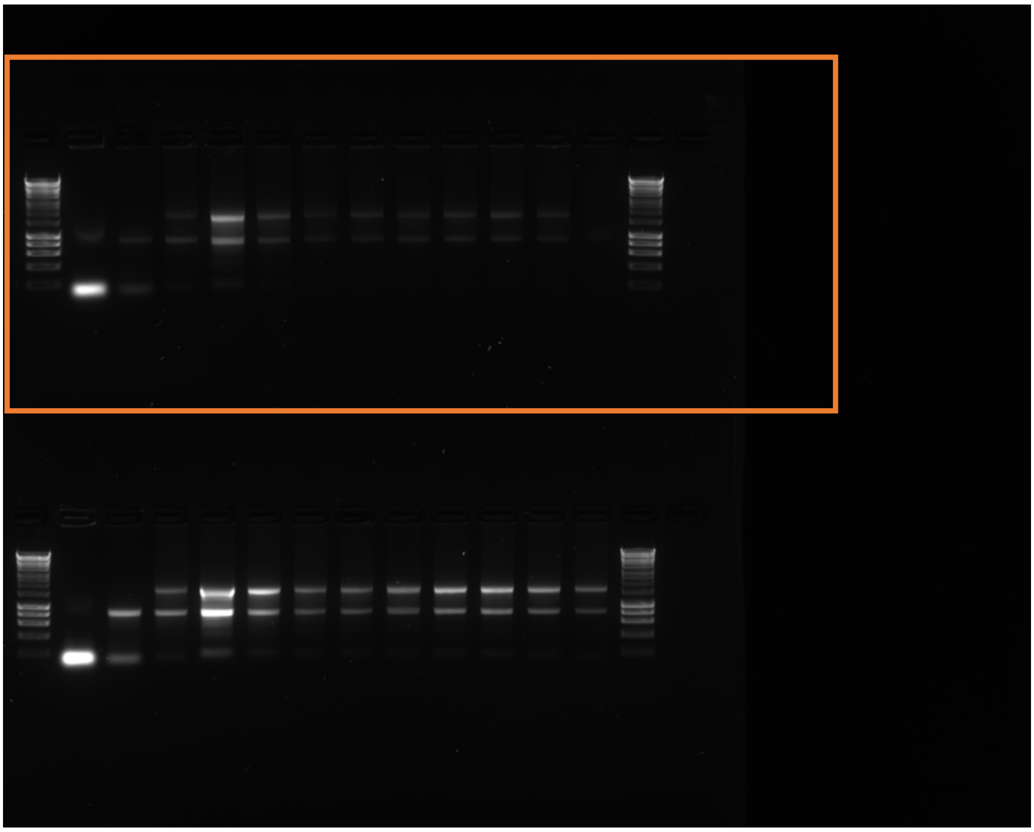

Main Fig. 4D

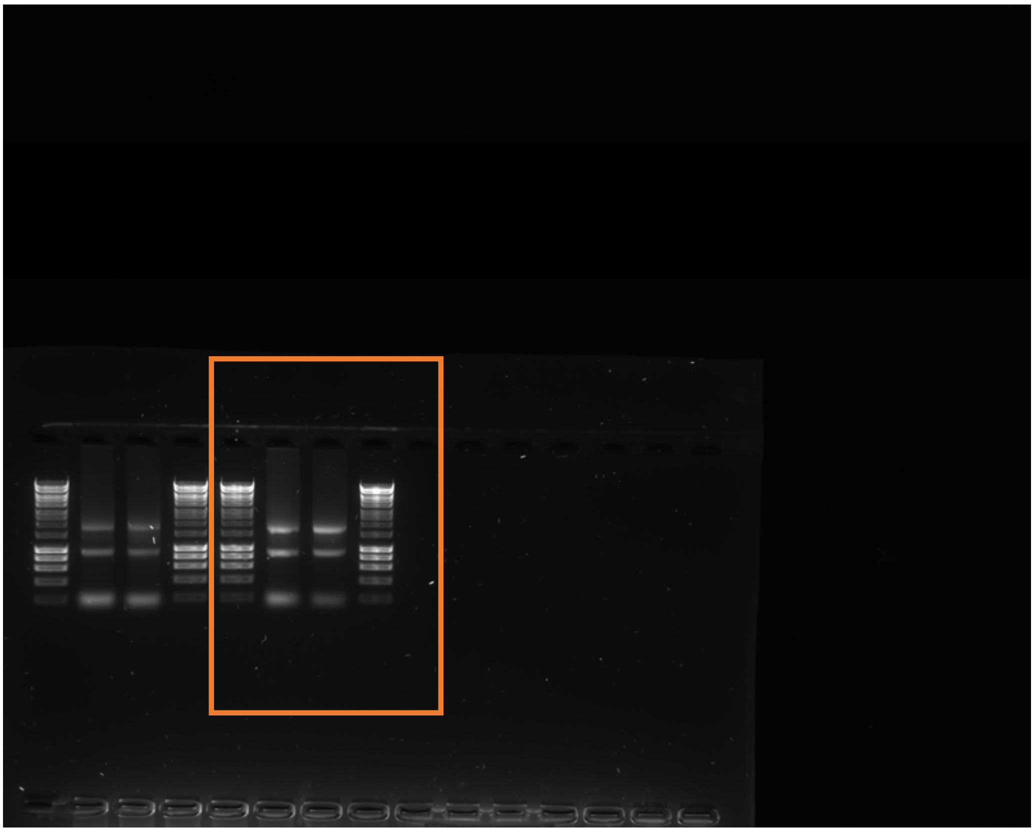

Main Fig. 4F

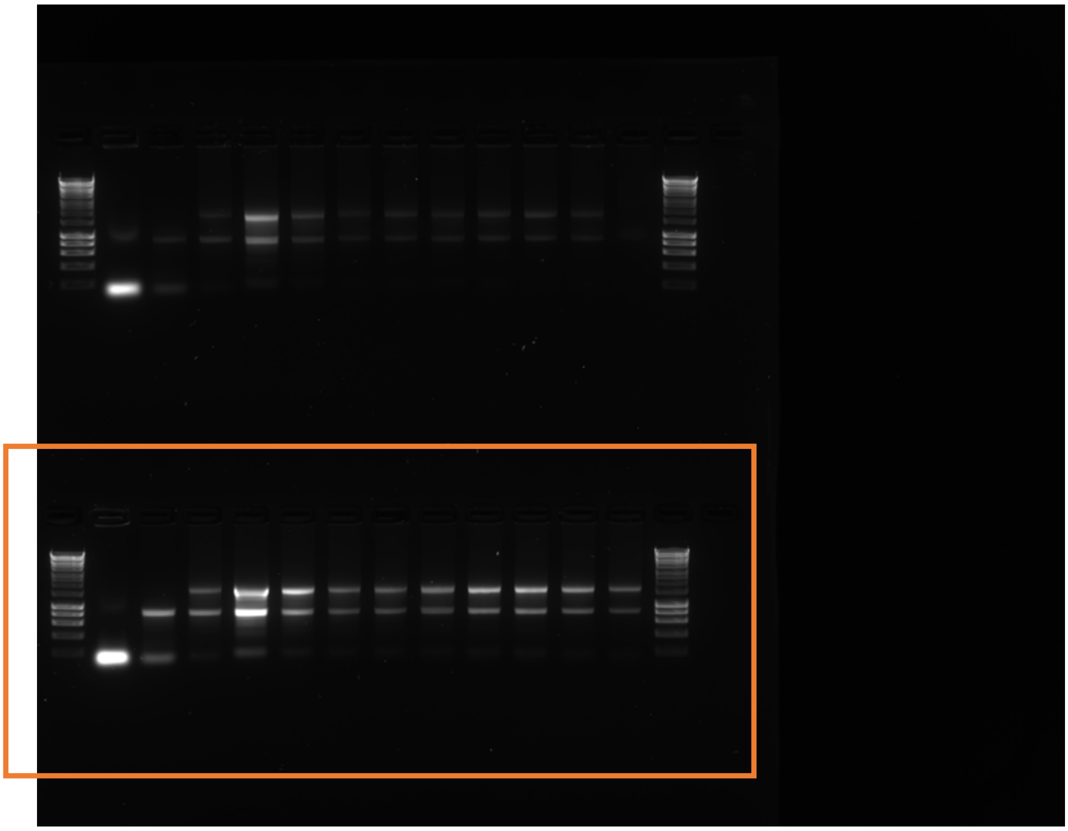

Main Fig. 5A

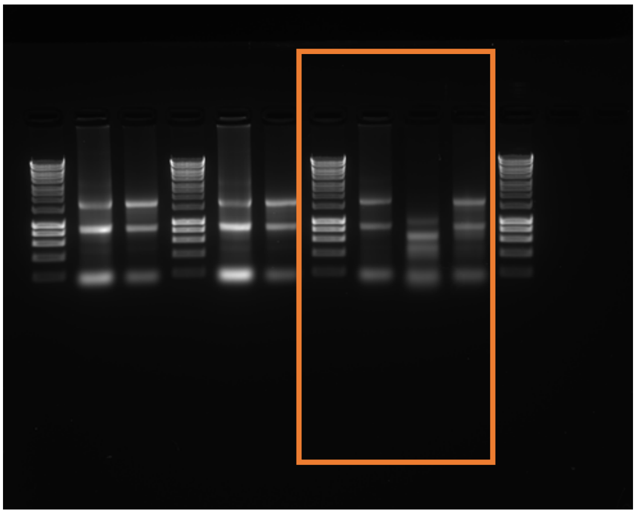

Main Fig. 5C

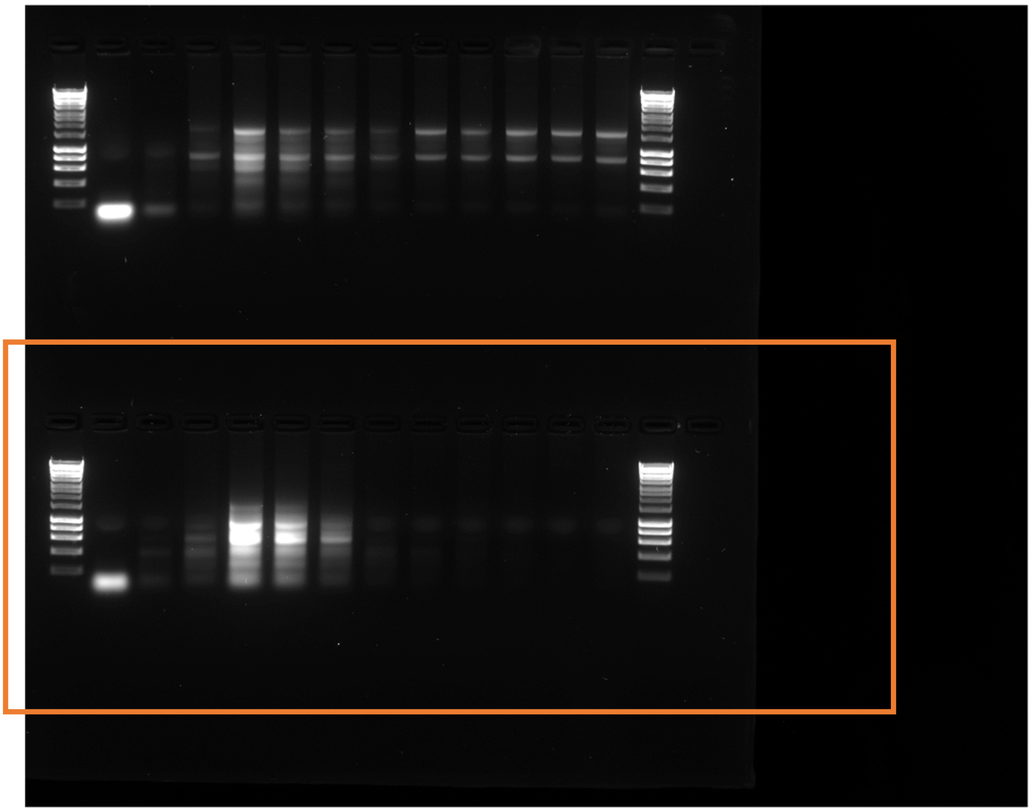

Main Fig. 5D

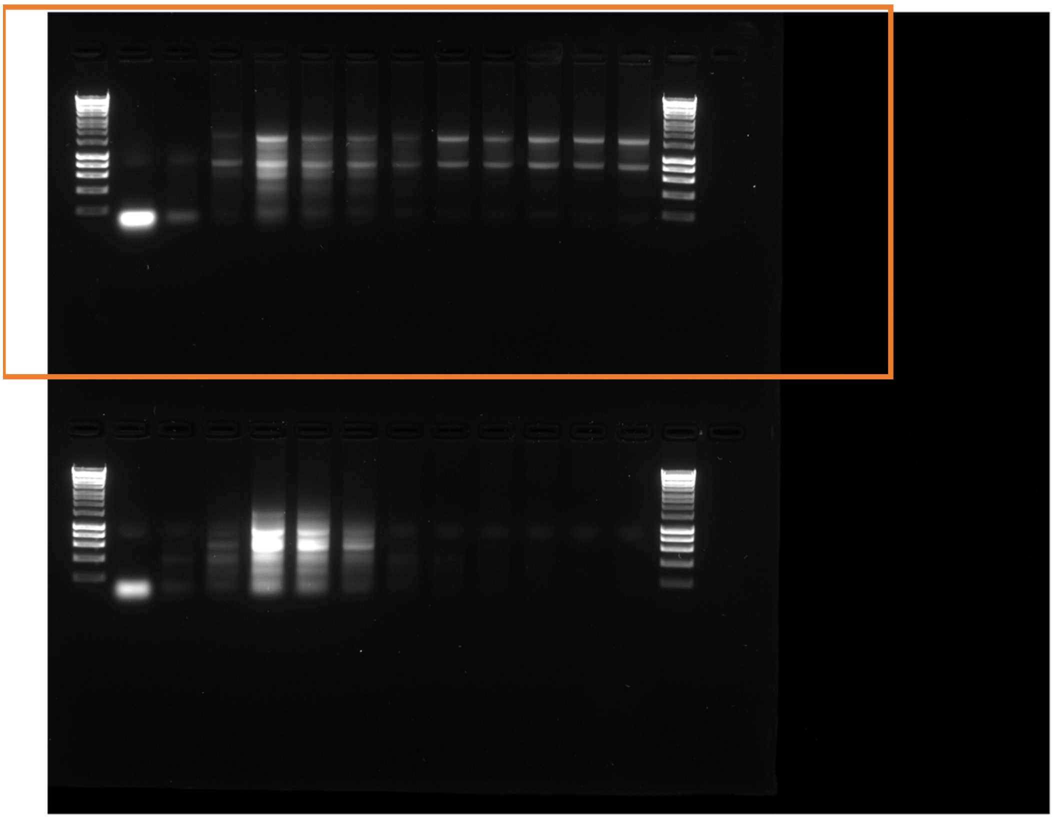

Supp. Fig 2A

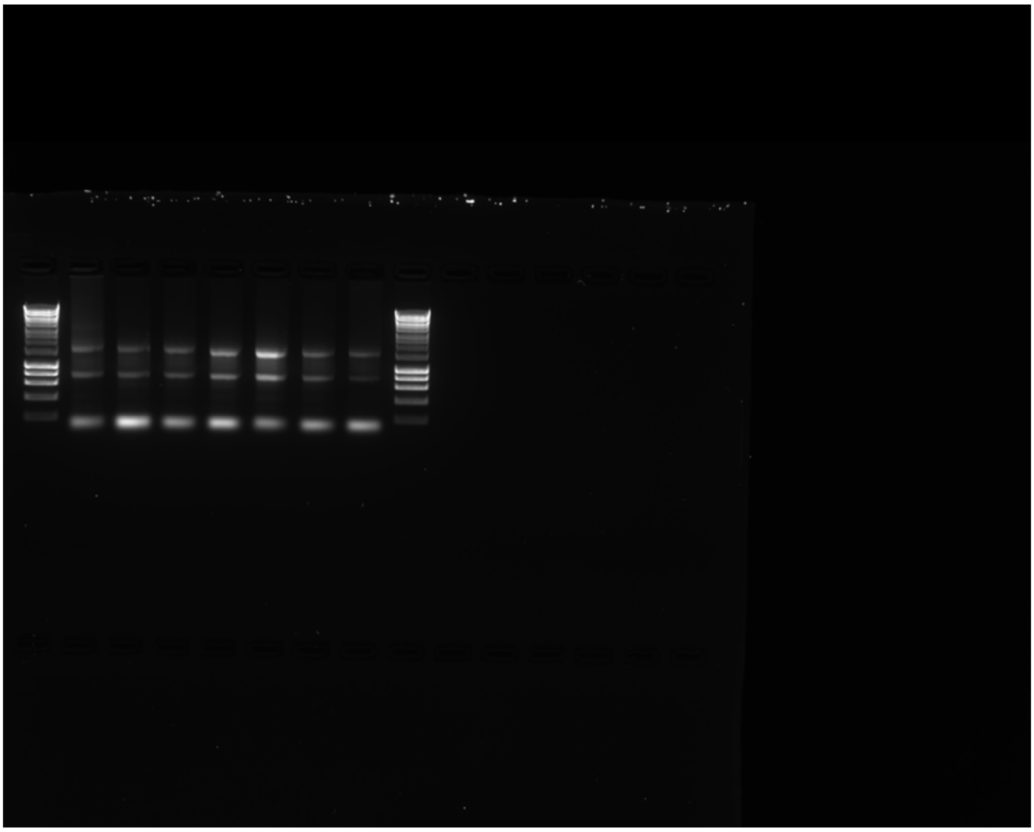

Supp. Fig 2B

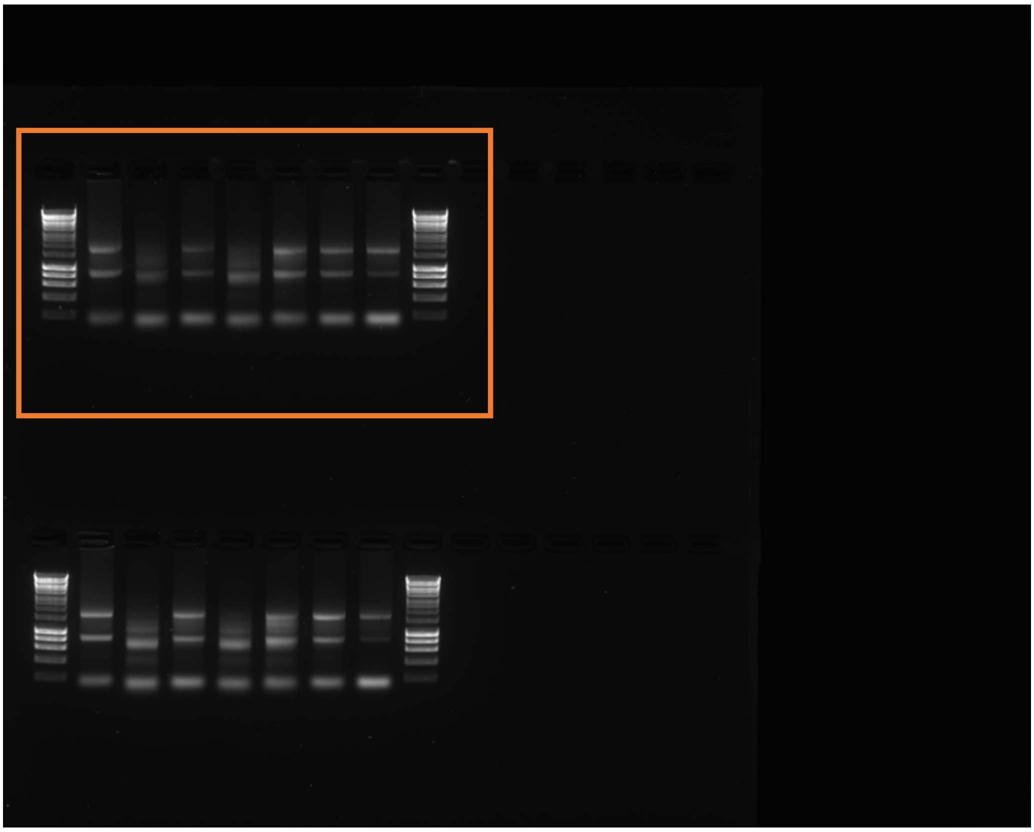

Supp. Fig 2C

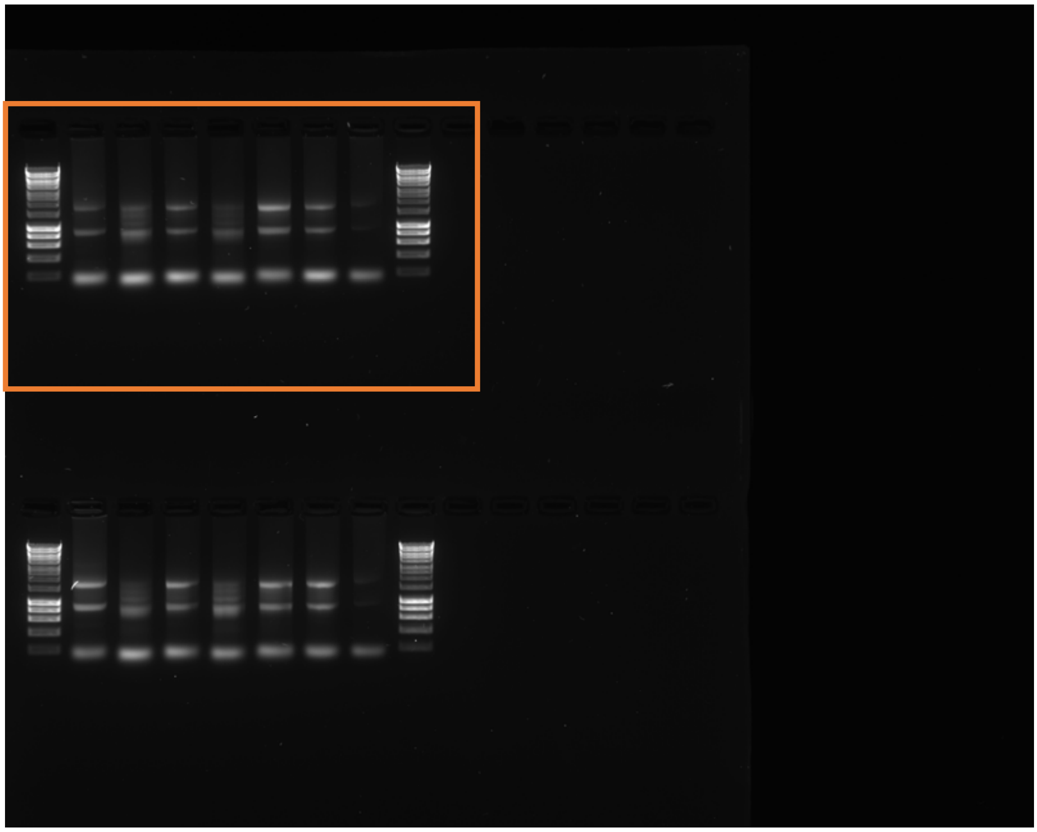

Supp. Fig 2D

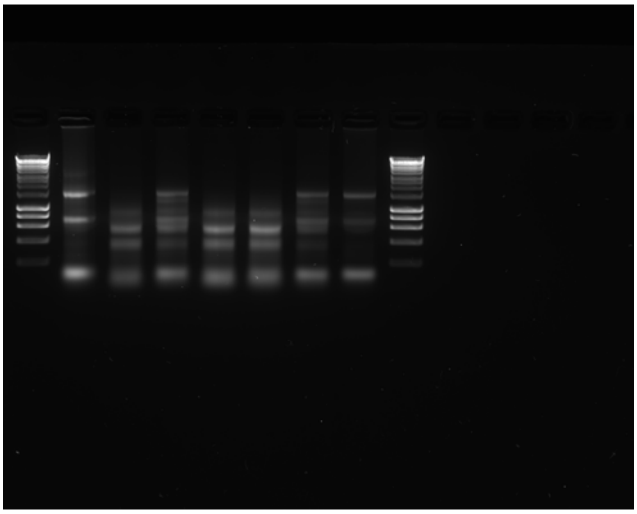

Supp. Fig 2E

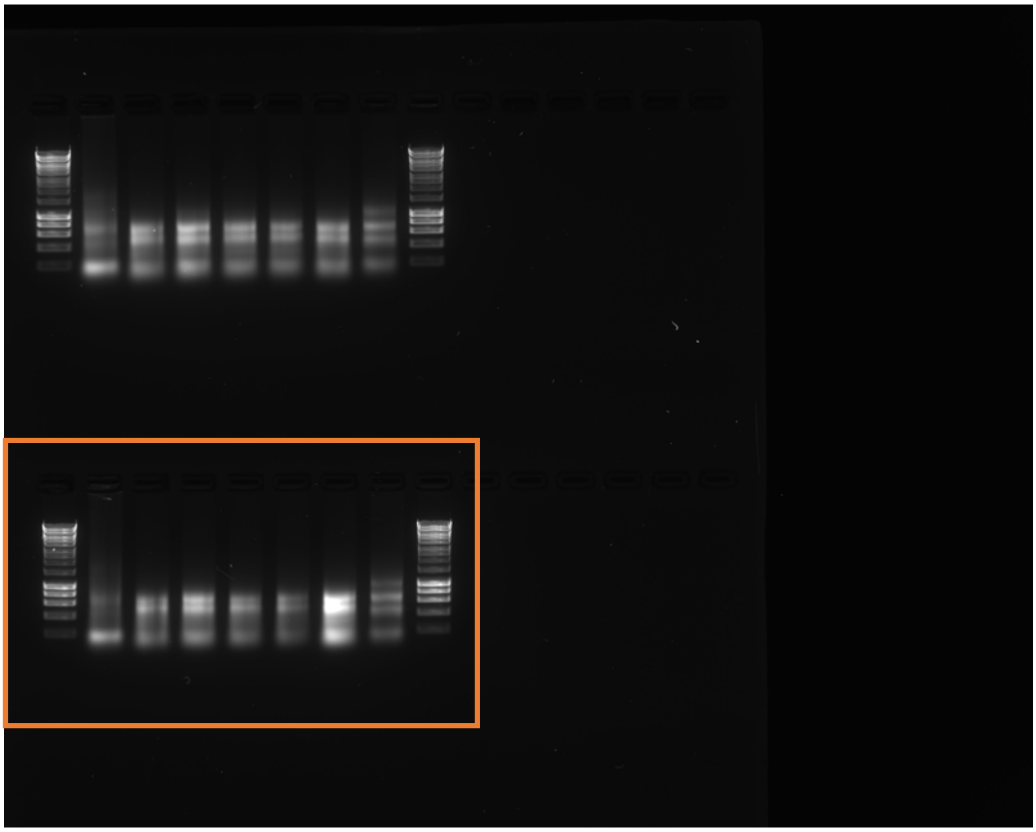

Supplementary Figure S3C

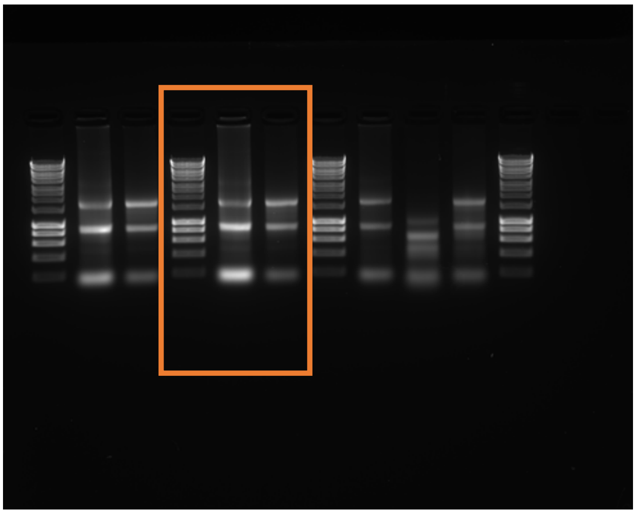

Supplementary Figure S3E

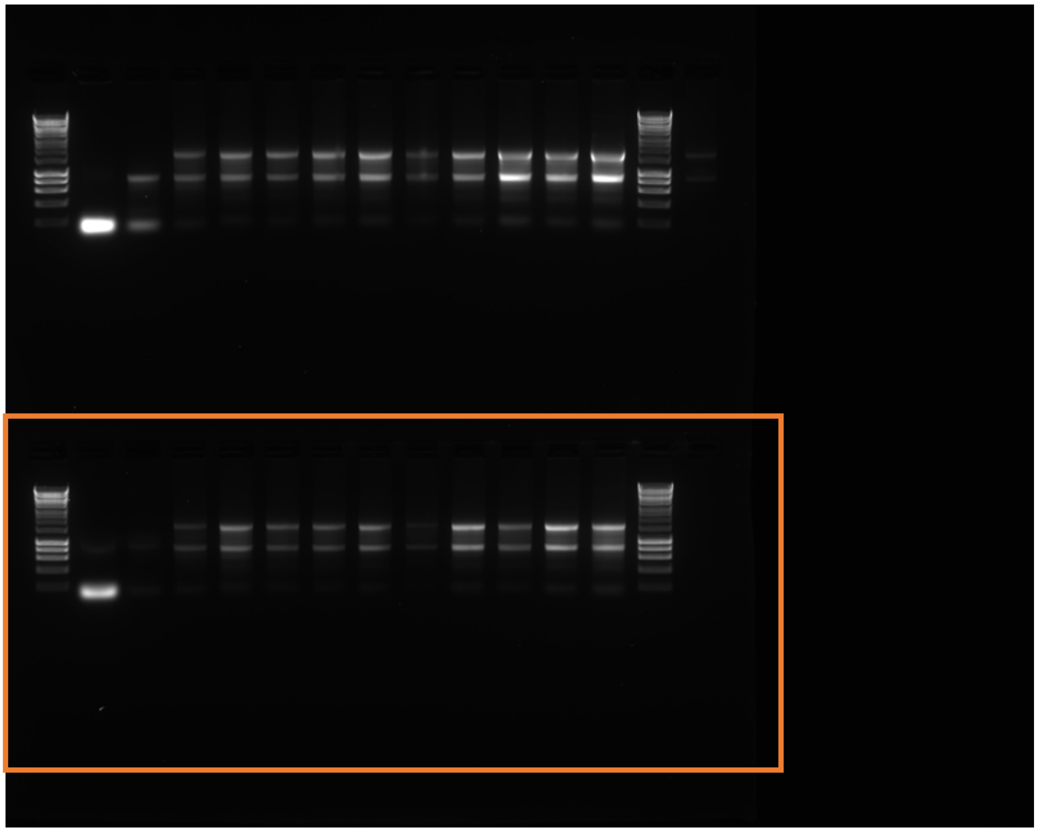

Supplementary Figure S4A

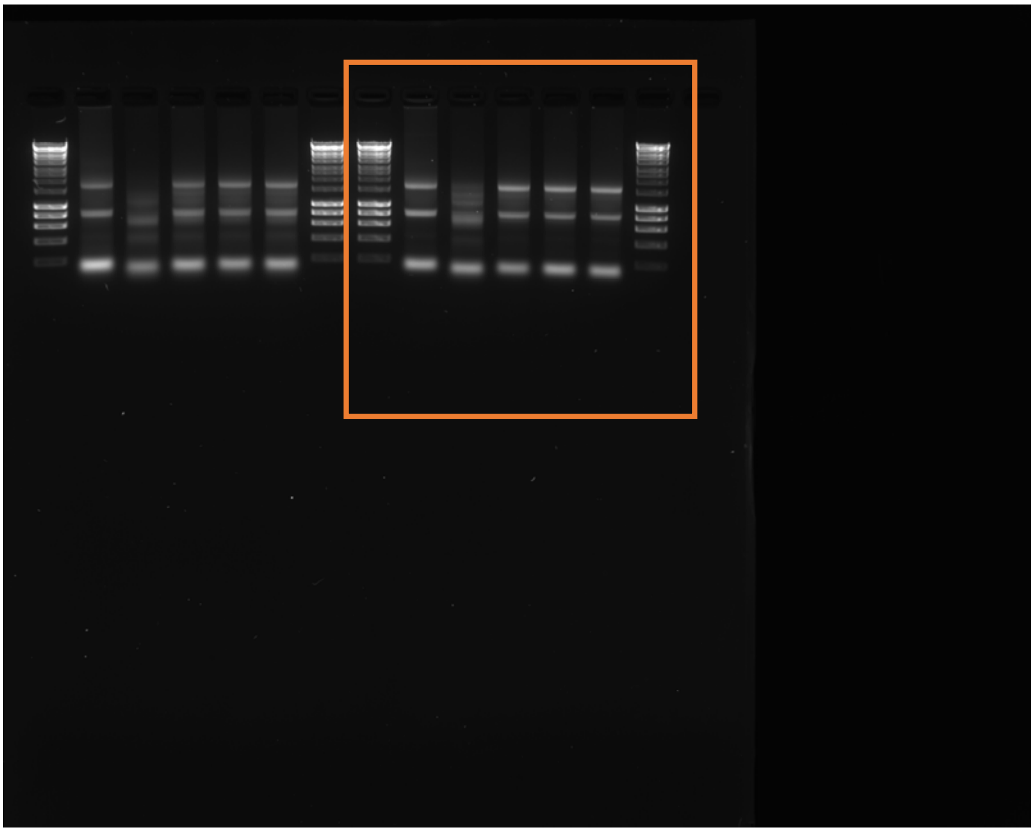

Supplementary Figure S4B

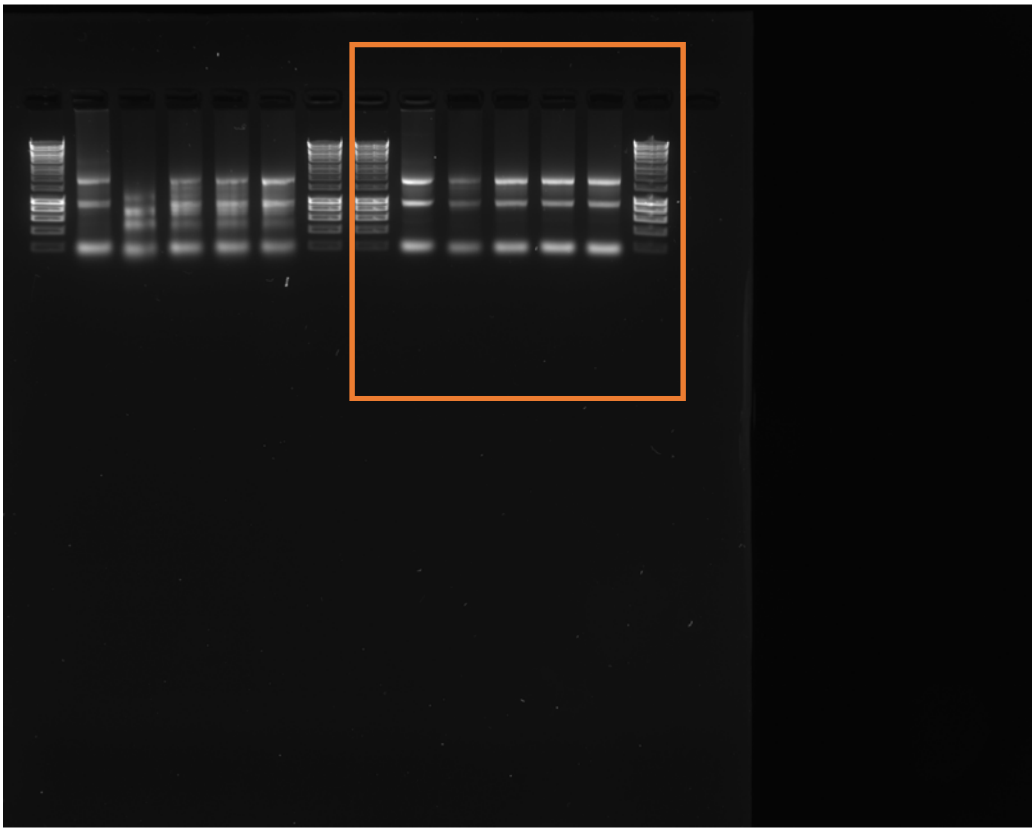

Supplementary Figure S4C

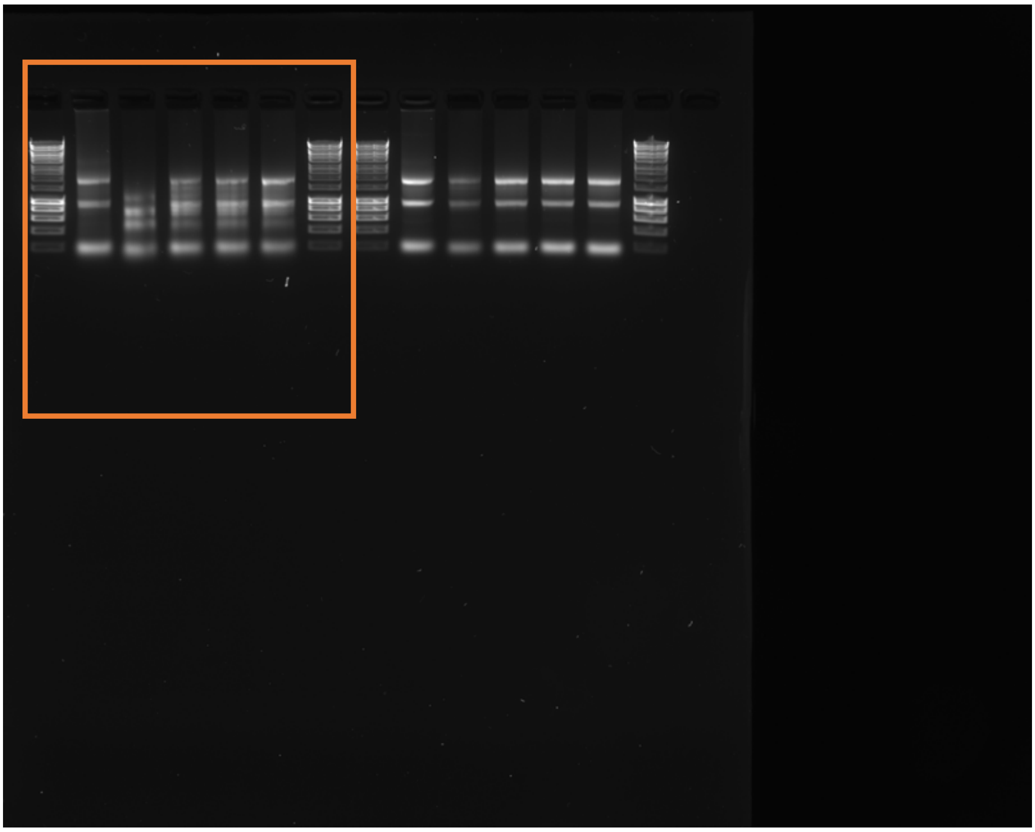

Supplementary Figure S4D

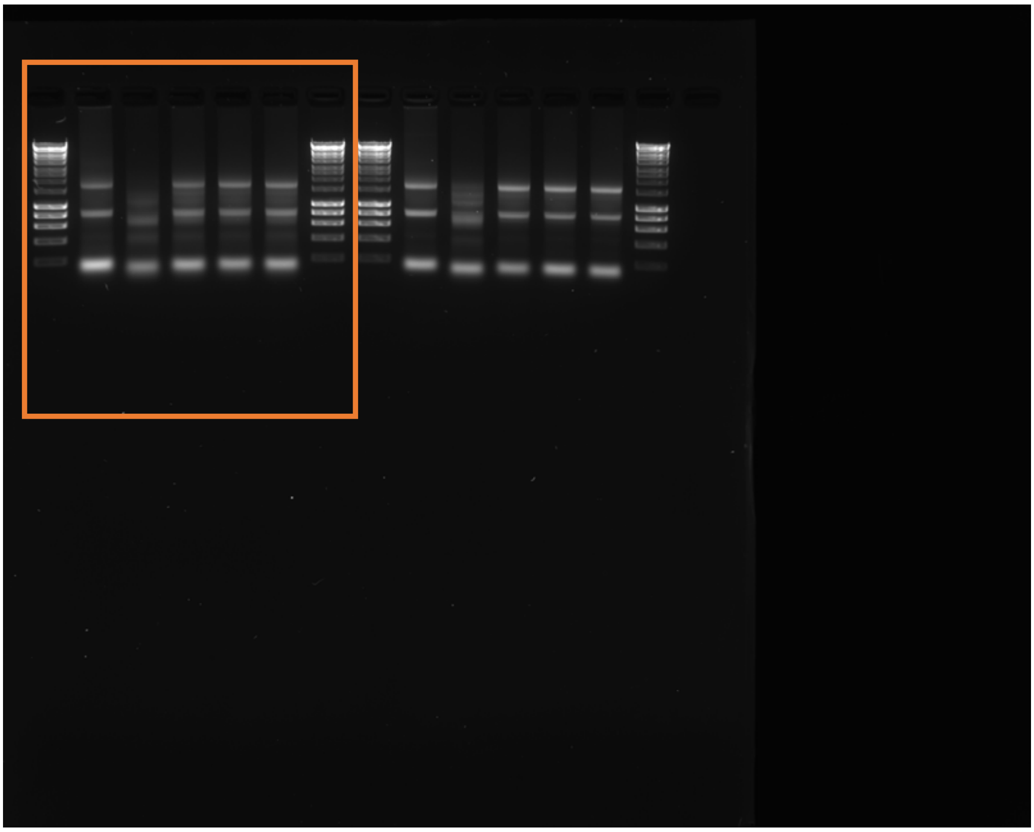

Supplementary Figure S5C

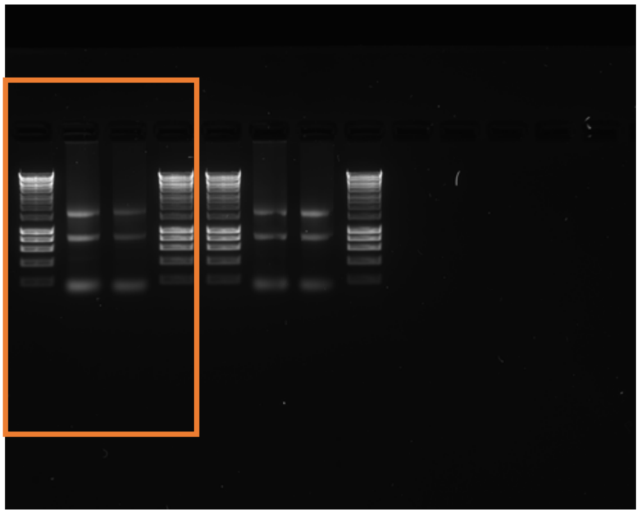

Supplementary Figure S5E

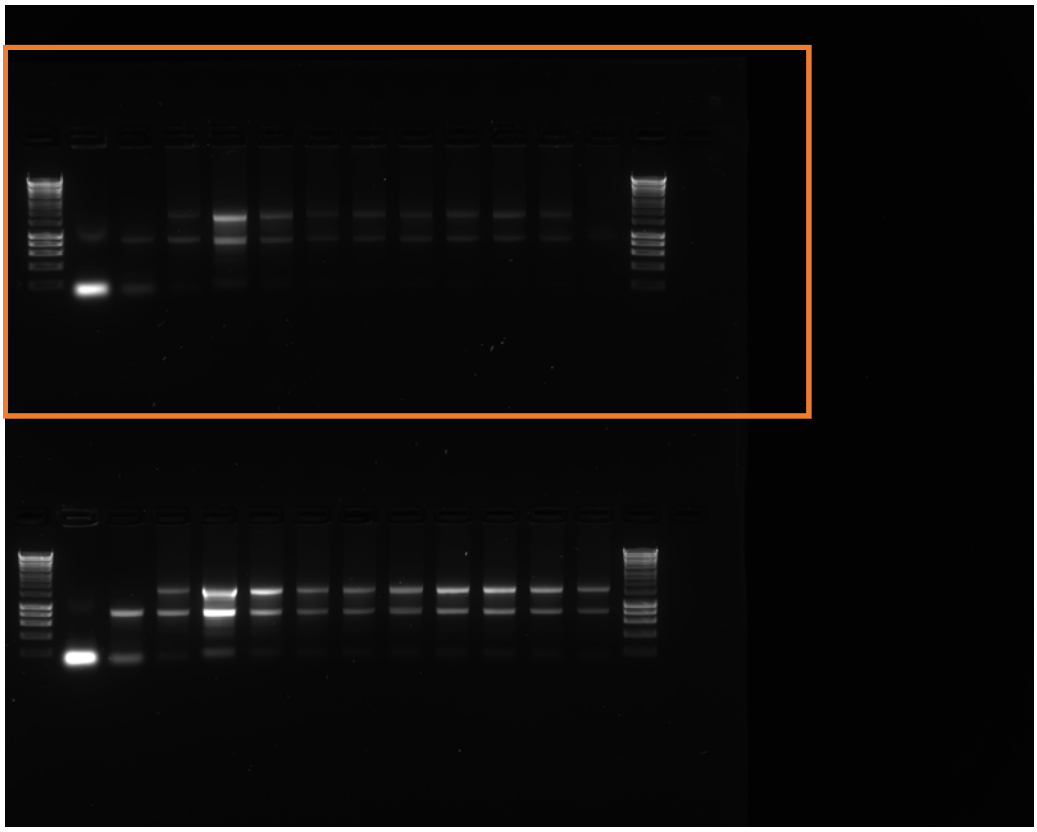

Supplementary Figure S5H

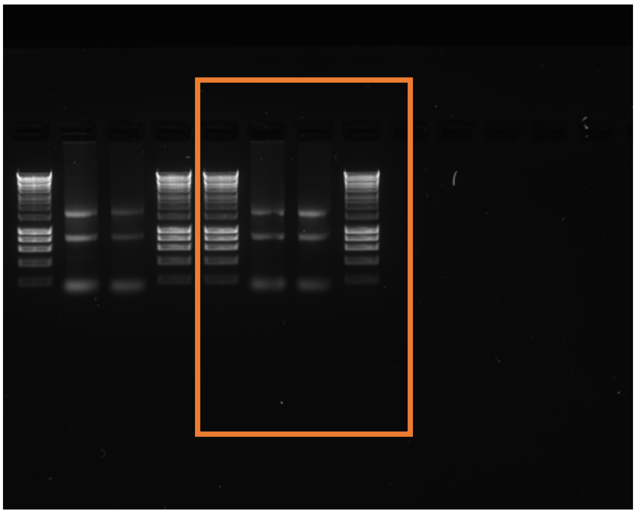

Supplementary Figure S5J

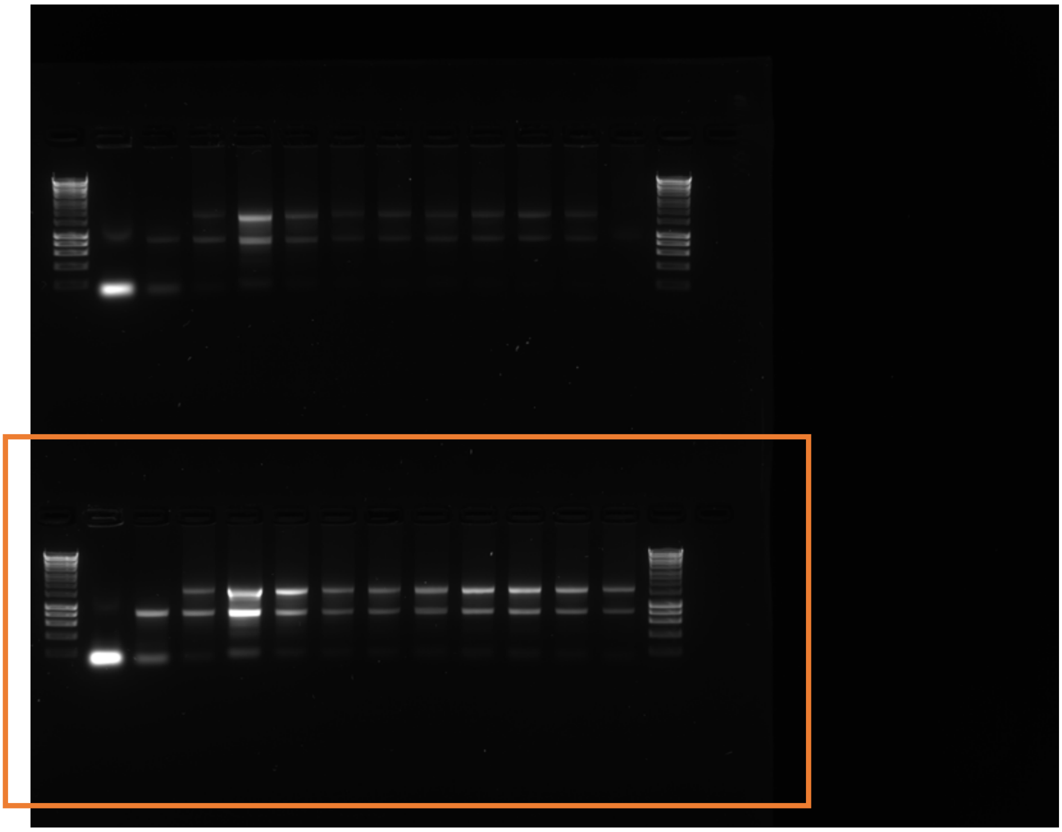

Supplementary Figure S6C

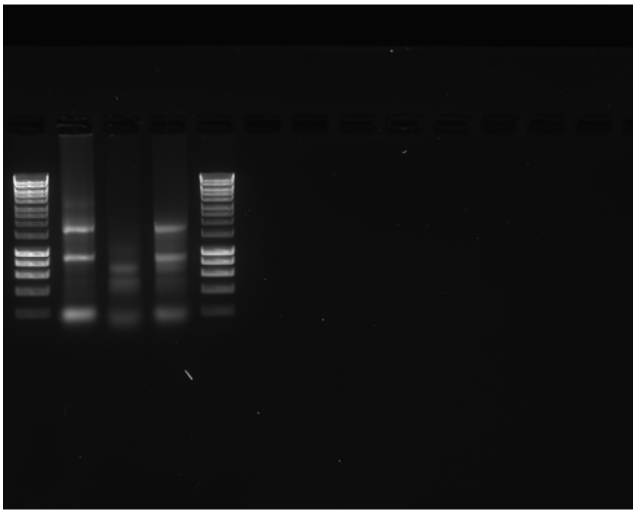

Supplementary Figure S6E

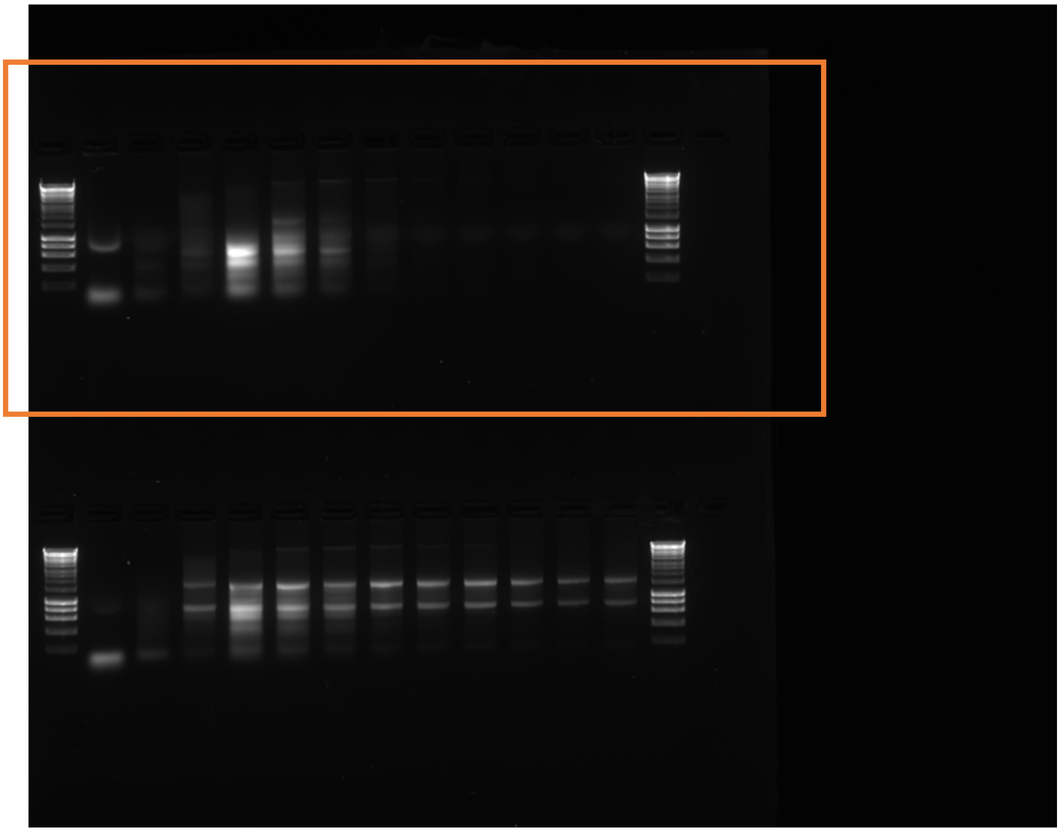

Supplementary Figure S6F

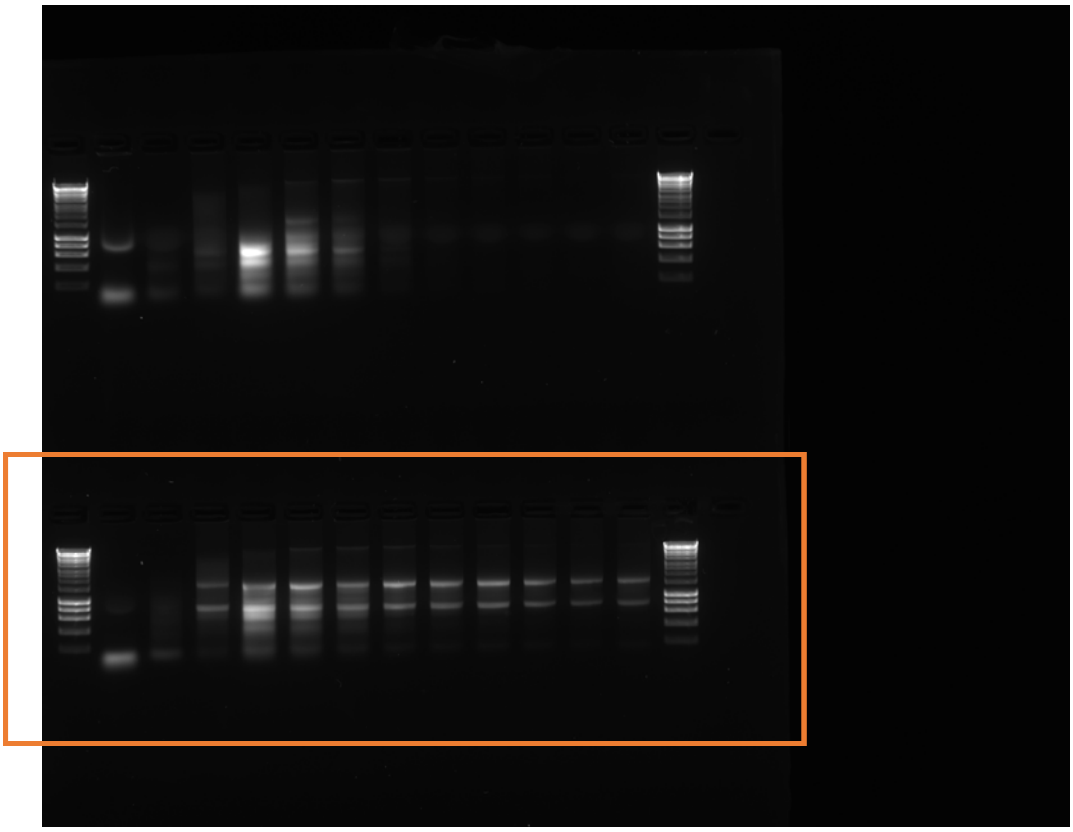

Supplement: Supplementary file 1 [file cancers-15-03985-s001.zip › cancers-2469282-File S1.pdf]
