# Supplementary material for: Optimisation of Sample Preparation from Primary Mouse Tissue to Maintain RNA Integrity for Methods Examining Translational Control
Source: Cancers (Basel). 2023 Aug 5;15(15):3985. doi: 10.3390/cancers15153985 (PMC10417042; doi:10.3390/cancers15153985)
Supplement: Supplementary file 1 [file cancers-15-03985-s001.zip › cancers-2469282-supplementary.pdf]

Supplementary Figure S1

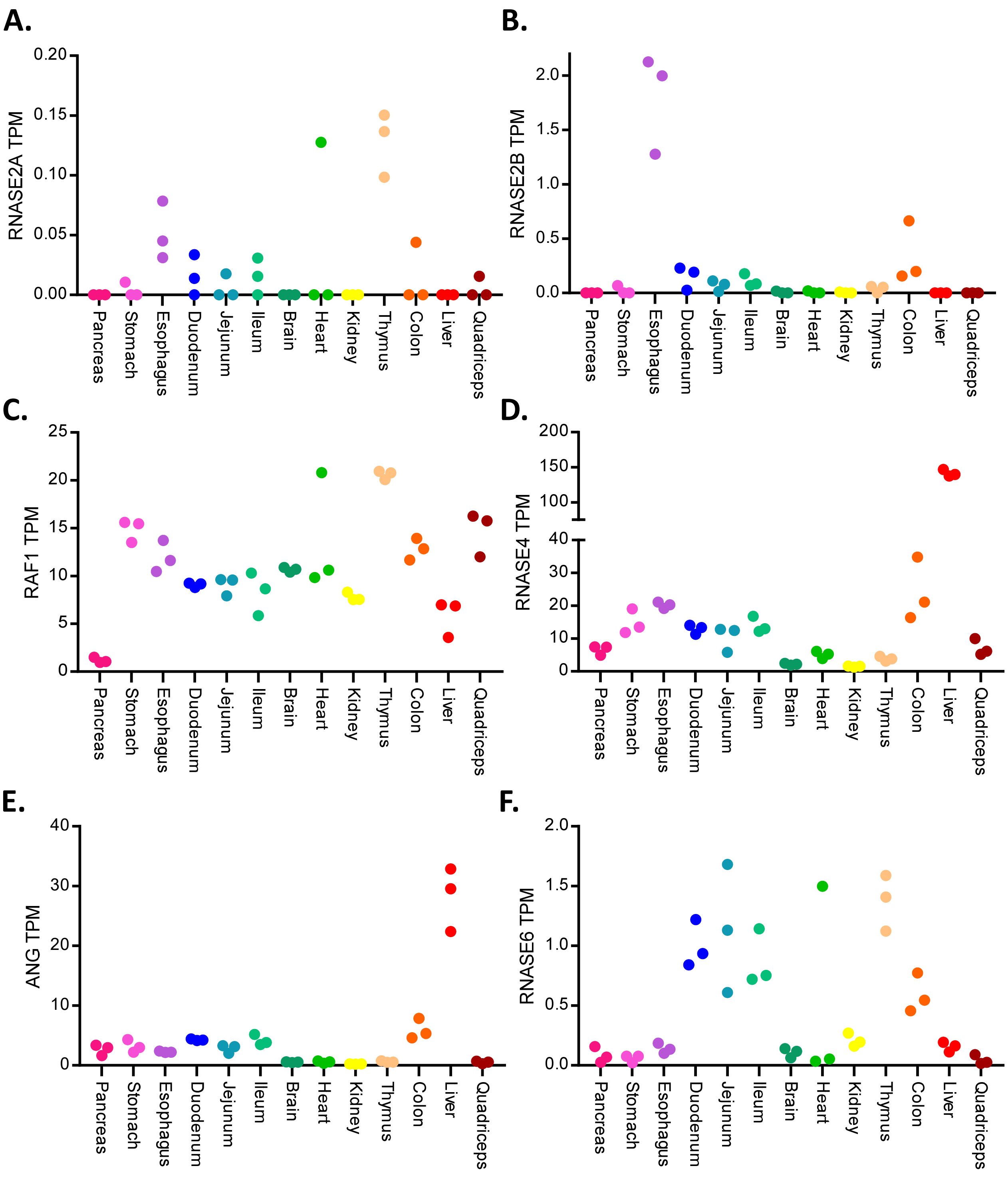

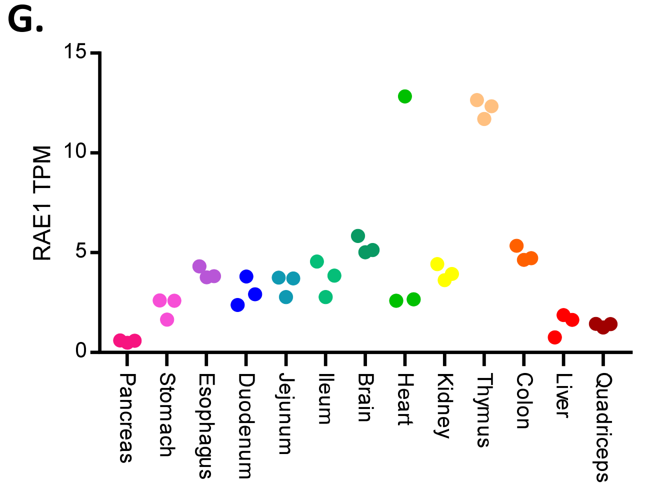

Supplementary Figure S1: Expression levels of RNases across mouse tissues. Expression levels (TPM: transcripts per million) of RNases and RNase inhibitors in several primary mouse tissues as determined using RNA-seq from Söllner et al, 2017. Each data point represents a replicate. (**A**) RNASE2A (**B**) RNASE2B (**C**) RAF1 (**D**) RNASE4 (**E**) ANG (**F**) RNASE6 (**G**) RAE1.

Supplementary Figure S2

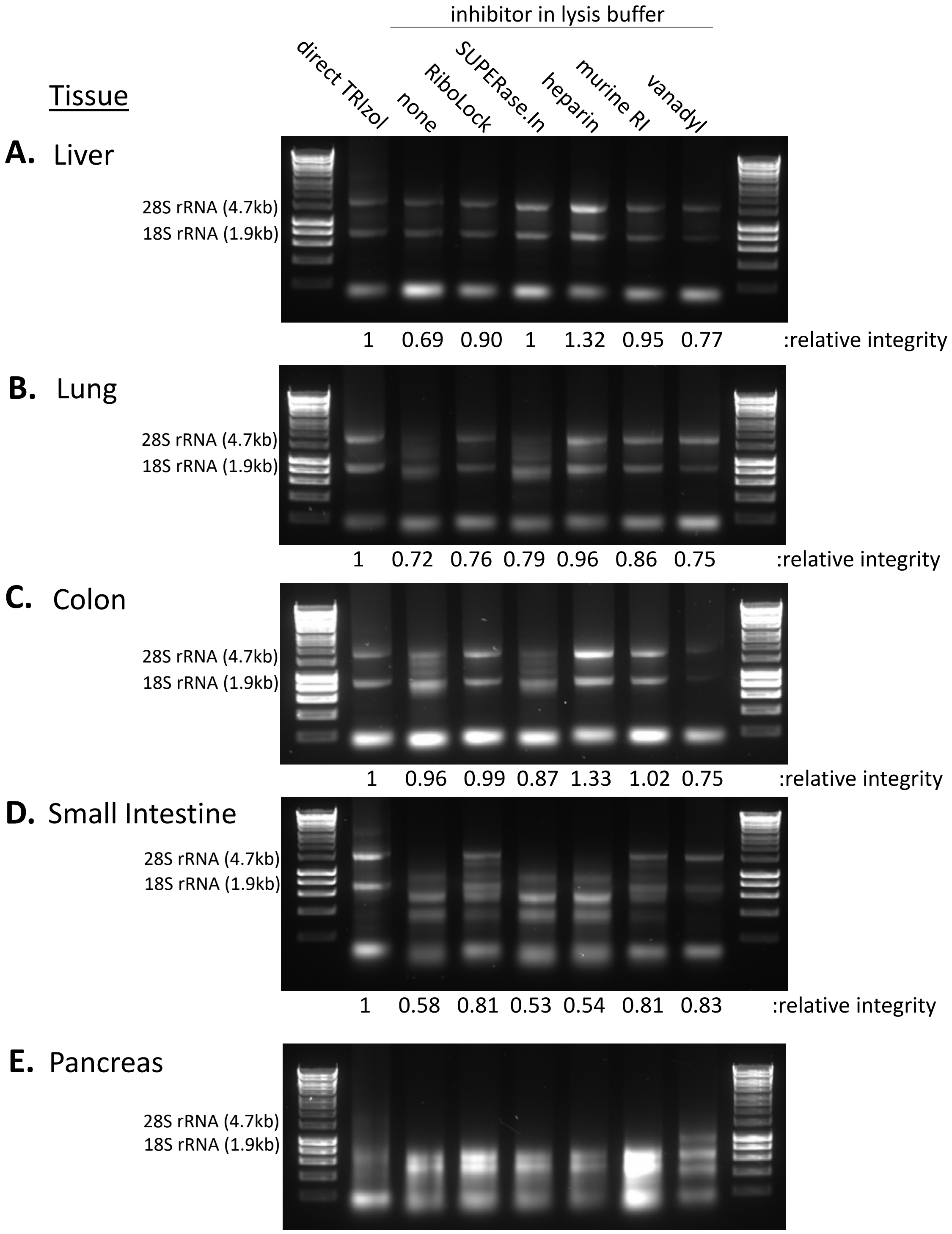

Supplementary Figure S2: RNA integrity across tissues under different non-denaturing lysis conditions repeat. (**A-E**) Additional biological replicate of RNA integrity for primary mouse tissues lysed in non-denaturing buffer containing one of a range of RNase inhibitors. Tissue harvested directly in TRIzol was used as a positive control for relative RNA integrity measures shown. Ladder used is 1kb HyperLadder. 28S rRNA and 18S rRNA sizes and expected positions are indicated. (**A**) Liver. (**B**) Lung. (**C**) Colon. (**D**) Small intestine. (**E**) Pancreas.

Supplementary Figure S3

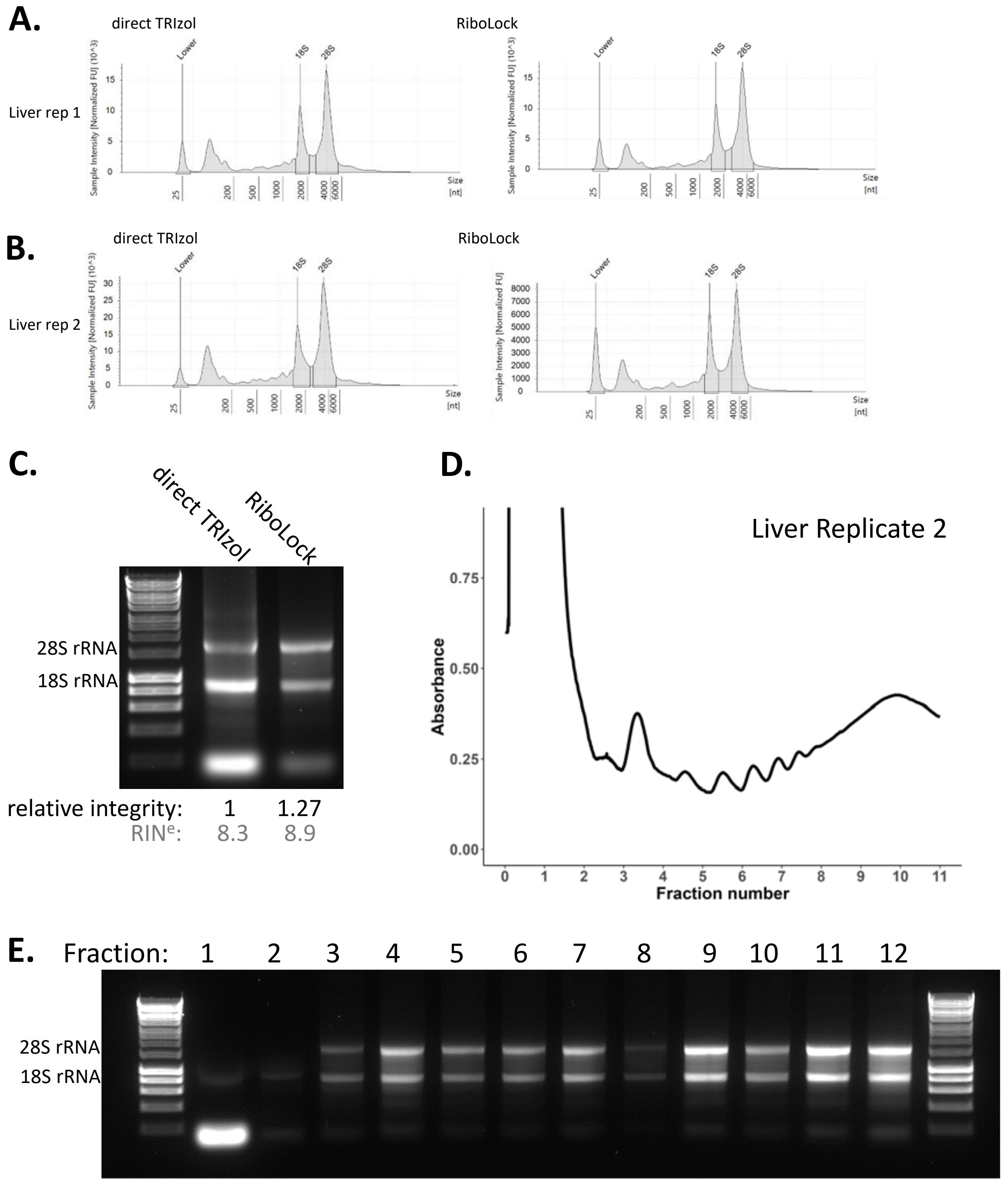

Supplementary Figure S3: Sucrose density gradients in a liver tissue repeat. (**A**) TapeStation traces for the total RNA in Fig. 3B. (**B-E**) Polysome profile in an independent biological replicate of mouse liver tissue. RNA integrity of liver tissue lysed directly in TRIzol and the total lysate, lysed in non-denaturing lysis buffer containing RiboLock, loaded on to the gradient. (**B**) TapeStation trace, (**C**) agarose gel with relative integrity and TapeStation RIN^e values shown (grey). (**D**) Polysome profile for mouse liver tissue. (**E**) RNA integrity across each of the individual gradient fractions.

Supplementary Figure S4

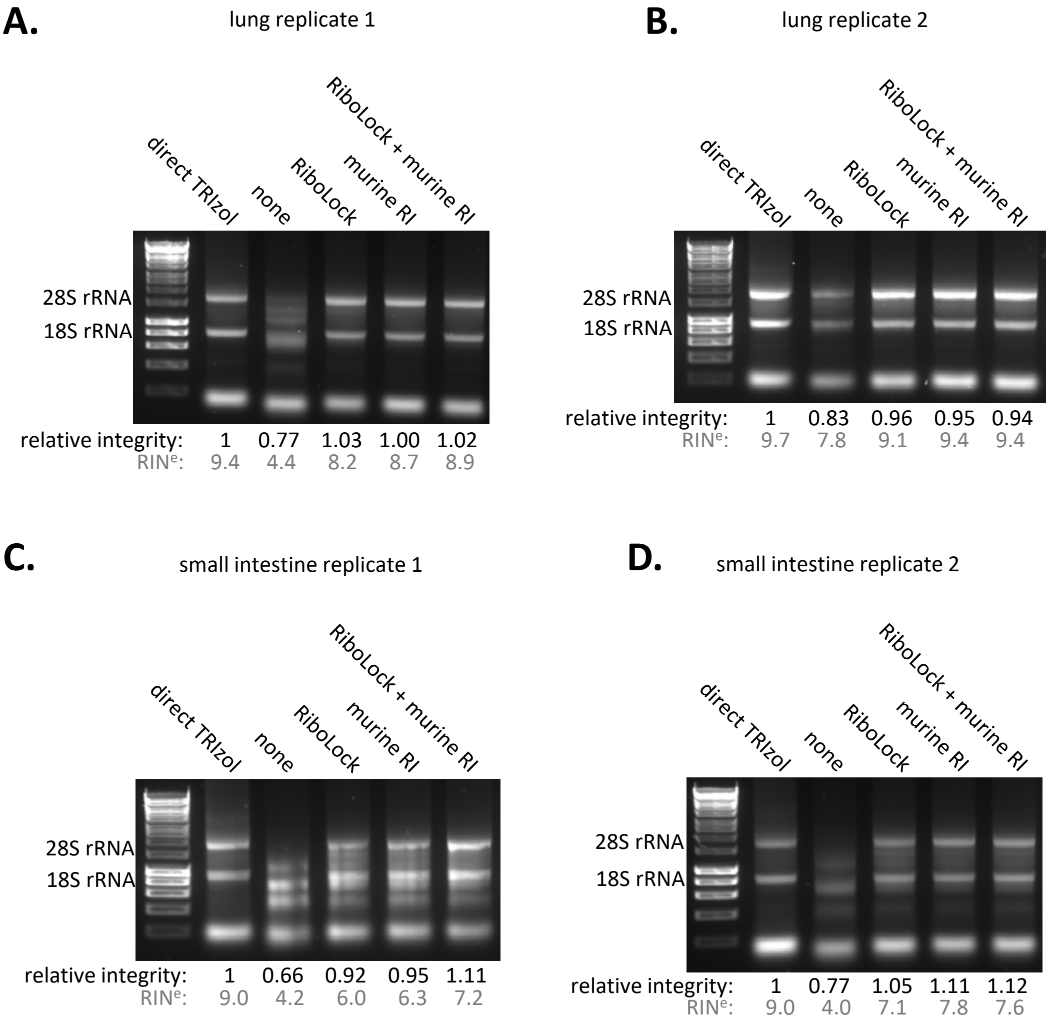

Supplementary Figure S4: Combinatorial RNase inhibitor testing. RNA integrity for primary mouse tissues lysed in non-denaturing lysis buffer containing no RNase inhibitors, RiboLock, murine RNase inhibitor or both RiboLock and murine RNase inhibitor. Agarose gels with relative integrity and TapeStation RIN^e values shown (grey). (**A**) & (**B**) two independent replicates in lung tissue. (**C**) & (**D**) two independent replicates in small intestine tissue.

Supplementary Figure S5

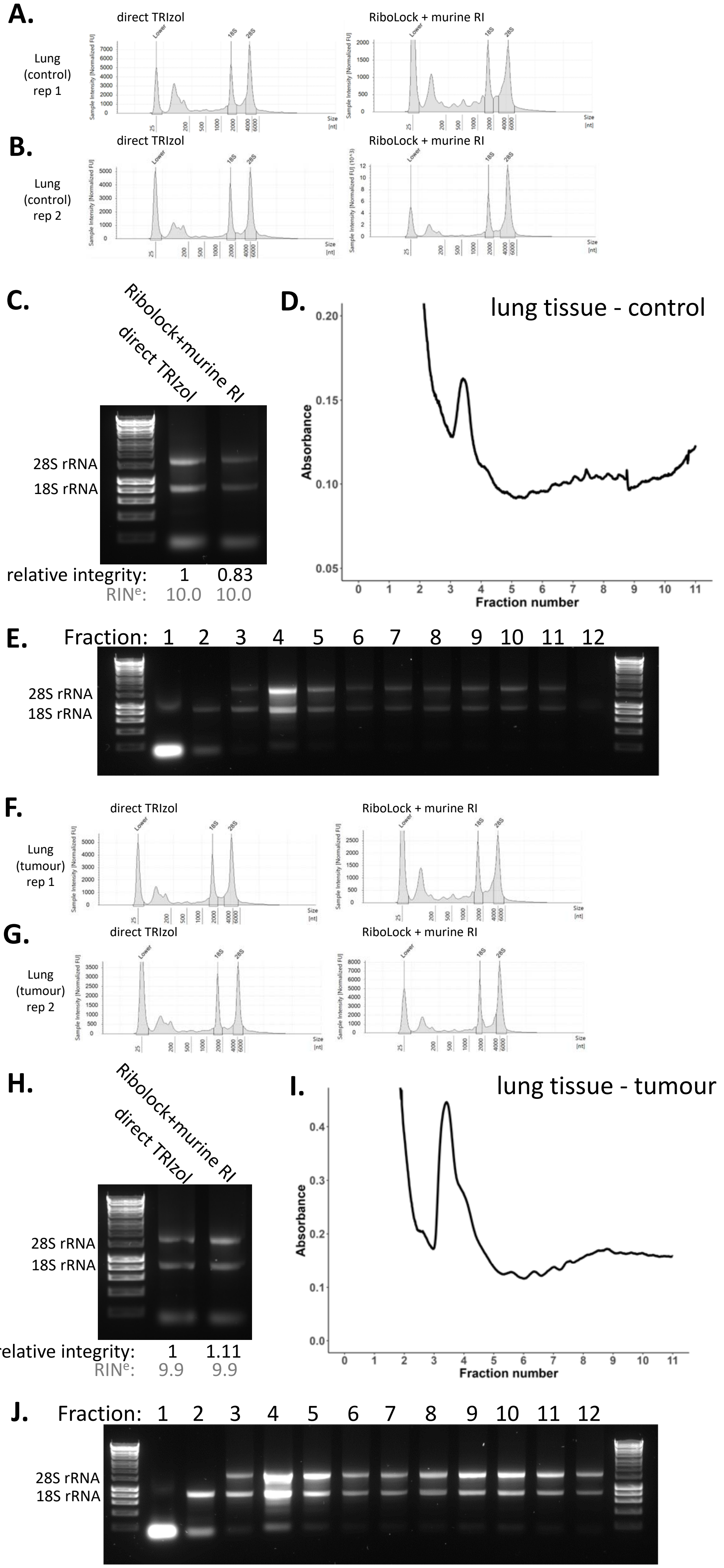

Supplementary Figure S5: Sucrose density gradients in a lung tissue replicate. (**A**) TapeStation traces for the total RNA in Fig. 4A. (**B-E**) Polysome profile in an independent biological replicate of mouse lung tissue. RNA integrity of lung tissue lysed directly in TRIzol and the total lysate, lysed in non-denaturing lysis buffer containing RiboLock loaded on to the gradient. (**B**) TapeStation traces, (**C**) agarose gel with relative integrity and TapeStation RIN^e values shown (grey). (**D**) Polysome profile for healthy mouse lung tissue as a control. (**E**) RNA integrity across each of the individual gradient fractions. (**F**) TapeStation traces for the total RNA in Fig. 4D. (**G-J**) Polysome profile in an independent biological replicate of mouse lung tumour tissue – pool of three tumour samples due to low amount of material. RNA integrity of lung tumour tissue lysed directly in TRIzol and the total lysate, lysed in non-denaturing lysis buffer containing RiboLock loaded on to the gradient. (**G**) TapeStation traces, (**H**) agarose gel with relative integrity and TapeStation RIN^e values shown (grey). (**I**) Polysome profile for mouse lung tumour tissue. (**J**) RNA integrity across each of the individual gradient fractions.

Supplementary Figure S6

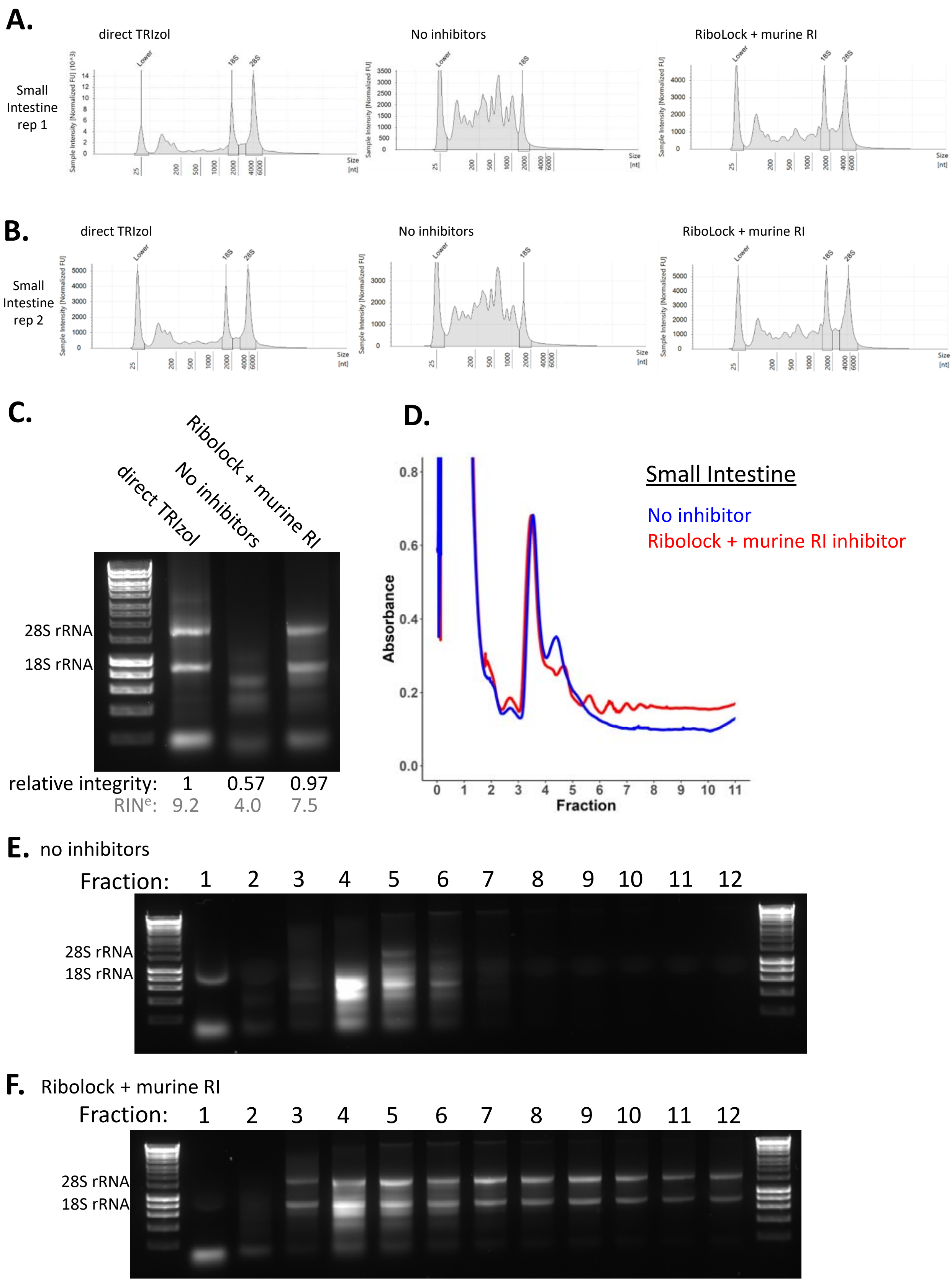

Supplementary Figure S6: Sucrose density gradients in a small intestine tissue repeat. (**A**) TapeStation traces for the total RNA in Fig. 5A. (**B-F**) Polysome profiles of mouse small intestine tissue in the presence and absence of RNase inhibitor for an additional biological replicate. RNA integrity of small intestine tissue lysed directly in TRIzol and the total lysate, lysed in non-denaturing lysis buffer without RNase inhibitor or containing a combination of RiboLock and murine RNase inhibited loaded on to the gradient. (**B**) TapeStation traces, (**C**) agarose gel with relative integrity and TapeStation RIN^e values shown (grey). (**D**) Polysome profile for mouse small intestine tissue, in the presence (red) and absence of (blue) RNase inhibitor. (**E**) RNA integrity across each of the individual gradient fractions for small intestine lysed in the absence of RNase inhibitors. (**F**) RNA integrity across each of the individual gradient fractions for small intestine lysed in the presence of a combination of RNase inhibitors RiboLock and murine RNase inhibitor.
